# Acute kidney injury in heart failure: a population study

**DOI:** 10.1002/ehf2.12595

**Published:** 2020-02-14

**Authors:** Jose Luis Holgado, Cristina Lopez, Antonio Fernandez, Inmaculada Sauri, Ruth Uso, Jose Luis Trillo, Sara Vela, Julio Nuñez, Josep Redon, Adrian Ruiz

**Affiliations:** ^1^ Cardiovascular and Renal Research Group, INCLIVA Research Institute University of Valencia Avda Blasco Ibañez, 17 46010 Valencia Spain; ^2^ Internal Medicine Hospital Clínico de Valencia Valencia Spain; ^3^ Cardiology Hospital Clínico de Valencia Valencia Spain; ^4^ CIBERObn Carlos III Health Institute Madrid Spain

**Keywords:** Heart failure, Acute kidney injury, Renal function, Risk of hospitalization, Risk of mortality

## Abstract

**Aims:**

The objective of the present study is to assess the prognostic value of acute kidney injury (AKI) in the evolution of patients with heart failure (HF) using real‐world data.

**Methods and results:**

Patients with a diagnosis of HF and with serial measurements of renal function collected throughout the study period were included. Estimated glomerular filtration rate (GFR) was calculated with the CKD‐EPI (Chronic Kidney Disease Epidemiology Collaboration). AKI was defined when a sudden drop in creatinine with posterior recovery was recorded. According to the Risk, Injury, Failure, Loss, and End‐Stage Renal Disease (RIFLE) scale, AKI severity was graded in three categories: *risk* [1.5‐fold increase in serum creatinine (sCr)], *injury* (2.0‐fold increase in sCr), and *failure* (3.0‐fold increase in sCr or sCr > 4.0 mg/dL). AKI incidence and risk of hospitalization and mortality after the first episode were calculated by adjusting for potential confounders. A total of 30 529 patients with HF were included. During an average follow‐up of 3.2 years, 5294 AKI episodes in 3970 patients (13.0%) and incidence of 3.3/100 HF patients/year were recorded. One episode was observed in 3161 (10.4%), two in 537 (1.8%), and three or more in 272 (0.9%). They were more frequent in women with diabetes and hypertension. The incidence increases across the GFR levels (Stages 1 to 4: *risk* 7.6%, 6.8%, 11.3%, and 12.5%; *injury* 2.1%, 2.0%, 3.3%, and 5.5%; and *failure* 0.9%, 0.6%. 1.4%, and 8.0%). A total of 3817 patients with acute HF admission were recorded during the follow‐up, with incidence of 38.4/100 HF patients/year, 3101 (81.2%) patients without AKI, 545 (14.3%) patients with one episode, and 171 (4.5%) patients with two or more. The number of AKI episodes [one hazard ratio (HR) 1.05 (0.98–1.13); two or more HR 2.01 (1.79–2.25)] and severity [*risk* HR 1.05 (0.97–1.04); *injury* HR 1.41 (1.24–1.60); and *failure* HR 1.90 (1.64–2.20)] increases the risk of hospitalization. A total of 10 560 deaths were recorded, with incidence of 9.3/100 HF patients/year, 8951 (33.7%) of subjects without AKI episodes, 1180 (11.17%) of subjects with one episode, and 429 (4.06%) with two or more episodes. The number of episodes [one HR 1.05 (0.98–1.13); two or more HR 2.01 (1.79–2.25)] and severity [*risk* 1.05 confidence interval (CI) (0.97–1.14), *injury* 1.41 (CI 1.24–1.60), and *failure* 1.90 (CI 1.64–2.20)] increases mortality risk.

**Conclusions:**

The study demonstrated the worse prognostic value of sudden renal function decline in HF patients and pointed to those with more future risk who require review of treatment and closer follow‐up.

## 1 Introduction

Heart failure (HF) is a mounting condition with huge impact in health care burden. Prevalence is still increasing mainly driven for the aging population in which renal dysfunction is also frequent.^1^ The association between HF and renal dysfunction is well known; while HF increases the risk of renal insufficiency, chronic kidney disease (CKD) increases the risk of hospitalization and mortality.^2^ Thus, the term ‘cardiorenal syndrome’ with various subtypes has been introduced.^3^

One of the cardiorenal syndromes is acute kidney injury (AKI), a syndrome of multiple aetiologies associated with increased risk of hospitalization and high mortality.^4^, ^5^ It has been recognized that even patients who have complete or near‐complete kidney function recovery after AKI are at increased risk of progressive CKD, and that superimposition of AKI on CKD is associated with acceleration in the rate of progression to end‐stage renal disease.^6^ In patients with HF, the incidence and impact of AKI have been reported mainly in subjects hospitalized with acute HF (AHF), in which the prevalence of AKI is ~20%, and it has been recognized that AKI is a strong independent predictor of both in‐hospital and 1‐year mortality.^7^, ^8^ A meta‐analysis of cohorts, registries, and post‐hoc studies concluded that CKD and worsening of renal function (WRF),^9^ a term that has been used instead of AKI in HF patients,^10^ are frequently observed in patients with HF. However, the authors acknowledge heterogeneity due to different inclusion criteria, selection bias, and different definitions and criteria used to qualify AKI.^9^ Indeed, the incidence, prevalence, and consequences of AKI in patients with HF are not well established.

The objective of the present study is to assess the prognostic value of AKI in the evolution of patients with HF using real‐world data. This study reflects the view that electronic health record (EHR)‐based studies from general practice are a representative setting to evaluate burden of disease associated with health conditions such as AKI in HF.

## 2 Subjects and methods

### 2.1 Study population and baseline data collection

The sample was recruited from beneficiaries of the Valencian Health Agency's universal health care system. The Valencian Community is a Mediterranean region located on the east coast of Spain, with a population of 3 799 885 people older than 18 years in 2012. Every patient has a unique personal identification number for the health system, so there is one unique electronic centralized clinical record per patient. The total population data were extracted using the health information exchange function of ABUCASIS for the period of time between 1 January 2012 and 31 December 2015. ABUCASIS includes information on patient demographics, medications, vital status, past medical history, and laboratory data, among others. Patients' data collected from the system during the study were documented by a process of pseudo‐anonymization, making it impossible to use this information to identify the patients because the only link between the data and the patient is a code not available to the researchers. The data generated during the study were handled according to the Spanish Law 3/2008 of Data Protection and Guaranty of Digital Rights the and corresponding European norms.^11^ The study was reviewed and approved by the Committee for Ethics and Clinical Trials of the Hospital Clinico of Valencia.

In the general population, 132 065 subjects were men and women with a diagnosis of HF (ICD *398.91*, *402.01*, *402.11*, *402.91*, *404.01*, *404.11*, *404.91*, *404.03*, *404.13*, *404.93*, and all *428*), and the eligible patients for the present study were those with serial measurements of renal function collected throughout the study period. Participants were included in the study from 1 January 2012 if they fulfilled the eligibility condition of an HF diagnosis before this time. Subsequently, participants newly diagnosed of HF during the study period until 31 December 2015 were also included. Finally, 30 529 subjects of both sexes aged 18 years or older who attended routine health examinations and fulfilled eligibility criteria were initially selected from the total population database. The observational study was undertaken as part of routine clinical practice.

### 2.2 Glomerular filtration rate and acute kidney injury

Serum creatinine was measured using a kinetic rate Jaffé method in a Hitachi Model 704 multichannel analyser (Boehringer Mannheim Diagnostics). Serum creatinine was calibrated to account for laboratory differences across time and to standardize to creatinine measures with isotope dilution mass spectrometry. Estimated glomerular filtration rate (eGFR) was calculated from calibrated creatinine, age, and sex by using the CKD‐EPI (Chronic Kidney Disease Epidemiology Collaboration)^12^, ^13^ and the KDIGO (Kidney Disease Improving Global Outcomes) stratification of eGFR^14^ at the baseline of the study in stable conditions, defined as no changes in creatinine levels in the previous 6 months. During the follow‐up, AKI was defined when a sudden drop in creatinine with posterior recovery at previous levels was recorded. According to the RIFLE scale,^15^ AKI severity was graded in three categories: *risk* [1.5‐fold increase in serum creatinine (sCr)], *injury* (2.0‐fold increase in sCr), and *failure* (3.0‐fold increase in sCr or sCr > 4.0 mg/dL). The number of episodes for each patient has been quantified and graded.

### 2.3 Cardiovascular risk factor definition

Body mass index (BMI) was calculated by dividing measured weight in kilograms by square of height in metres. Obesity was defined as a BMI ≥ 30 kg/m^2^. Blood pressure was measured up to three times on the same day in a sitting position, and hypertension was defined as an office mean systolic blood pressure ≥ 140 mmHg, a mean diastolic blood pressure ≥ 90 mmHg, a recorded physician diagnosis, or medication use. Diabetes was defined as a non‐fasting glucose ≥ 200 mg/dL, a recorded physician diagnosis, medication use or an HbA1c ≥ 6.5%. Serum total cholesterol was measured enzymatically using the Cholesterol High Performance reagent (Roche Diagnostics). High‐density lipoprotein (HDL) cholesterol was measured using a direct HDL reagent (Roche Diagnostics). Low‐density lipoprotein cholesterol was calculated by using the Friedewald formula. Dyslipidaemia was defined by total cholesterol > 200 mg/dL and/or treatment with lipid‐lowering drugs.

### 2.4 Mortality and hospitalization follow‐up

Participants were followed up for hospitalization for AHF and for all‐cause mortality until 31 December 2015. Causes of hospitalization were recorded using codes of the *International Classification of Diseases, 9th Revision*. Vital status was determined by matching records and death certificates from the Spanish National Death Index. Mortality included all causes of death. Time to event was calculated for each individual as the difference between the date of the inclusion into the study and the date of the hospital admission, the date of death, or 31 December 2015, whichever occurred first.

### 2.5 Statistical analysis

The cumulative survival rates and HF events in each of the groups (presence of CKD or AKI and the degree of AKI) were analysed using Kaplan–Meier curves, and the log‐rank test was used to calculate the statistical significance of the differences. The prognostic value of the eGFR groups or AKI was assessed using a Cox regression hazard model to determine the hazard ratio (HR) for risk of hospitalization and mortality. Clinically relevant factors affecting the prognosis, including age (continuous modelled as restricted cubic splines with 5 knots), sex (men or women), BMI (continuous), hypertension (no or yes), diabetes (no or yes), angiotensin AT1 receptor blockers (ARBs), angiotensin‐converting‐enzyme inhibitors (ACEi), anti‐aldosterone drugs, and diuretics (Henle loop and thiazides), were selected for inclusion in the multivariate analysis. Multivariate Cox regression hazard model was performed by using the backward stepwise selection.

## 3 Results

### 3.1 General characteristics of the study population

A total of 30 529 patients with HF were included. Mean age was 75 years, and 58% were female. Hypertension was present in 89.4%, dyslipidaemia in 65.0%, and diabetes in 48.3% of the participants. A total of 12 809 (42.0%) patients had eGFR < 60 mL/min/1.73 m^2^, among them 35.4% eGFR between 30 and 60 mL/min/1.73 m^2^ and 6.5% between 15 and 30 mL/min/1.73 m^2^. The number of subjects with one episode of AKI was 3161 (10.4%) and two or more in 809 (2.6%). The main characteristics of the study population grouped by the number of AKI episodes are shown in *Table* 1. Patients with AKI episodes were more frequently female with diabetes and hypertension. Likewise, significant lower eGFR values were observed across the AKI severity groups. The treatments are also shown in *Table* 1. Patients with AKI were receiving more diuretics, beta‐blockers, and renin–angiotensin aldosterone system blockers (ARB, ACEi, and anti‐aldosterone drugs).

**Table 1:** General characteristics of the study population

	All subjects	No AKI	AKI risk	AKI injury	AKI failure
Number	30 529	26 559	2712	821	437
Sex (M)	14 030 (46.0)	12 173 (45.8)	1232 (45.4)^a^	379 (46.2)^a^	246 (56.3)^a^^,^ b ^,^ c
Body mass index (kg/m^2^)	30.8 (5.8)	30.8 (5.8)	30.8 (5.6)	31.0 (5.7)	30.4 (6.5)
Age at diagnosis	75.1 (11.0)	75.1 (11.1)	75.4 (10.2)	75.9 (9.9)	74.6 (10.7)
eGFR (mL/min/1.73 m^2^)	65.0 (22.4)	66.0 (21.9)	59.8 (23.4)^a^	58.2 (24.7)^a^	46.5 (27.0)^a^^,^ b ^,^ c
CKD Stage 1, >90	13.4	13.8	11.4^a^	10.6^a^	8.2^a^^,^ b ^,^ c
CKD Stage 2, 90–60	44.6	46.4	34.3	33.3	19.9
CKD Stage 3, 60–30	35.4	34.2	45.1	42.9	35.5
CKD Stage 4, 30–15	6.5	5.6	9.2	13.3	36.4
Average of visits^d^	19.4 (19.1)	18.9 (18.2)	23.8 (23.1)^a^	21.2 (24.6)^a^	22.1 (26.1)^a^
Acute HF hospitalization	3817 (12.5)	3101 (11.7)	472 (17.4)^a^	171 (20.8)^a^^,^ b	73 (16.7)^a^
Mortality	10 560 (34.6)	8951 (33.7)	992 (36.6)	367 (44.7)^a^^,^ b	250 (57.2)^a^^,^ b ^,^ c
Co‐morbidities					
Anaemia	16 145 (52.9)	13 534 (51.0)	1760 (64.9)^a^	541 (65.9)^a^	310 (70.9)^a^
Diabetes	14 740 (48.3)	12 484 (47.0)	1536 (56.6)^a^	475 (57.9)^a^	245 (56.1)^a^
Dyslipidaemia	19 831 (65.0)	17 135 (64.5)	1861 (68.6)^a^	545 (66.4)	290 (66.4)
Hypertension	27 303 (89.4)	23 595 (88.8)	2526 (93.1)^a^	767 (93.4)^a^	415 (95.0)^a^
Myocardial infarction	5569 (18.2)	4695 (17.7)	604 (22.3)^a^	174 (21.2)^a^	96 (22.0)
Atrial fibrillation	17 159 (56.2)	14 675 (55.3)	1689 (62.3)^a^	541 (65.9)^a^	254 (58.1)^c^
Treatment					
Diuretics	22 715 (74.4)	19 471 (73.3)	2232 (82.3)^a^	663 (80.8)^a^	349 (79.9)^a^
Beta‐blockers	13 131 (43.0)	11 199 (42.2)	1326 (48.9)^a^	406 (49.5)^a^	200 (45.8)
ACEi/ARB	19 859 (65.0)	17 053 (64.2)	1924 (70.9)^a^	596 (72.6)^a^	286 (65.4)
Calcium antagonists	7795 (25.5)	6569 (24.7)	797 (29.4)^a^	258 (31.4)^a^	171 (39.1)^a^^,^ b ^,^ c
NSAIDs	8660 (28.4)	7668 (28.9)	711 (26.2)^a^	191 (23.3)^a^	90 (20.6)^a^
Anti‐aldosterone	5477 (17.9)	4537 (17.1)	631 (23.3)^a^	216 (26.3)^a^	93 (21.3)

ACEi, angiotensin‐converting‐enzyme inhibitor; AKI, acute kidney injury; ARB, angiotensin receptor blocker; CKD, chronic kidney disease; eGFR, estimated glomerular filtration rate; NSAIDs, non‐steroidal anti‐inflammatory drug.

Values are number (percentage).

a Difference with no AKI group.

b Difference with AKI *risk* group.

c Difference with AKI *injury* group.

d Visits to specialists and primary care physicians.

### 3.2 Acute kidney injury episodes

**Figure 1:**
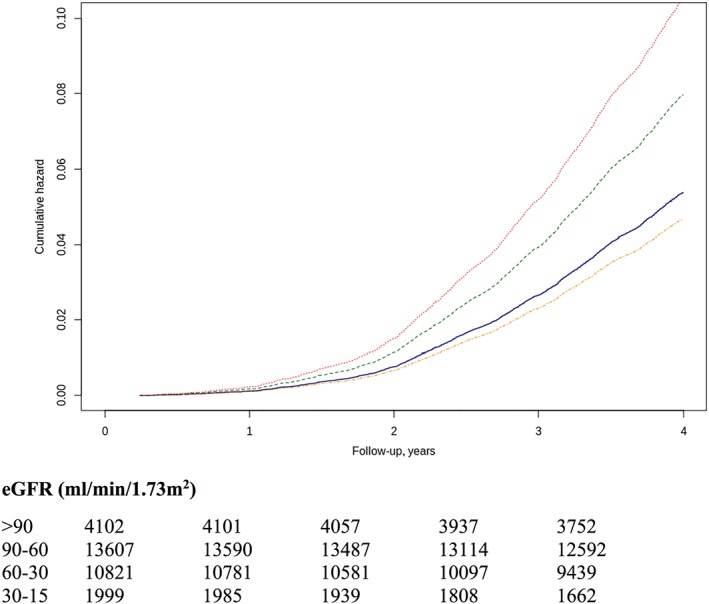
Risk to develop AKI episodes by CKD stage. Using as a reference the group in CKD Stage 1, the risk of *risk* (A) was HR 0.87 (95% CI 0.76–0.99) in Stage 2, HR 1.48 (95% CI 1.29–1.69) in Stage 3, and HR 1.95 (95% CI 1.65–2.30) in Stage 4. *Injury* (B) was HR 0.97 (95% CI 0.76–1.25) in Stage 2, HR 1.58 (95% CI 1.22–2.05) in Stage 3, and HR 3.49 (95% CI 2.61–4.66) in Stage 4. *Failure* (C) was HR 1.09 (95% CI 0.72–1.63) in Stage 2, HR 2.75 (95% CI 1.84–4.11) in Stage 3, and HR 16.36 (95% CI 10.84–24.69) in Stage 3. Lines: blue (Stage 1), orange (Stage 2), green (Stage 3), and red (Stage 4). AKI, acute kidney injury; CI, confidence interval; CKD, chronic kidney disease; HR, hazard ratio.

During an average follow‐up of 3.2 years, 5294 episodes of AKI in 3970 patients (13%), with incidence of 3.3/100 patients/year, were recorded. Only one episode was observed in 3161 (10.4%), two in 537 (1.8%), and three or more in 272 (0.9%). The odds ratio of a second episode after the first was 0.26 (95% CI 0.24–0.28). According to the severity, AKI *risk* was present in 2712 patients, *injury* in 821, and *failure* in 437. The prevalence increases across the reduction of GFR levels (Stages 1 to 4: *risk* 7.6%, 6.8%, 11.3%, and 12.5%; *injury* 2.1%, 2.0%, 3.3%, and 5.5%; and *failure* 0.9%, 0.6%. 1.4%, and 8.0%, respectively) and incidence rate (Stages 1 to 4: *risk* 2.0, 1.8, 3.2, and 3.7/100 patients/year; *injury* 0.5, 0.5, 0.9, and 1.6/100 patients/year; and *failure* 0.2, 0.2, 0.4, and 2.3/100 patients/year, respectively) (*Figures* *1* *and* *2*).

**Figure 2:**
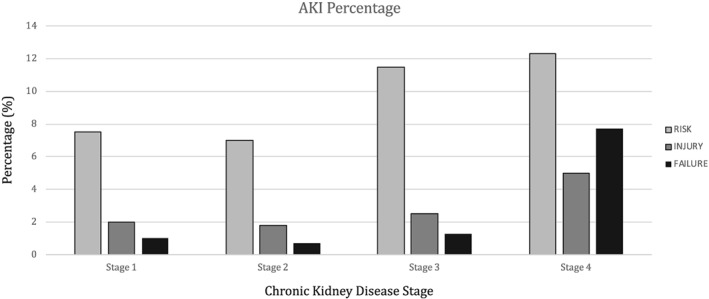
Percentage of patients with AKI severity episodes by CKD stage. AKI, acute kidney injury; CKD, chronic kidney disease.

### 3.3 Acute kidney injury episodes and acute heart failure hospitalization

**Figure 3:**
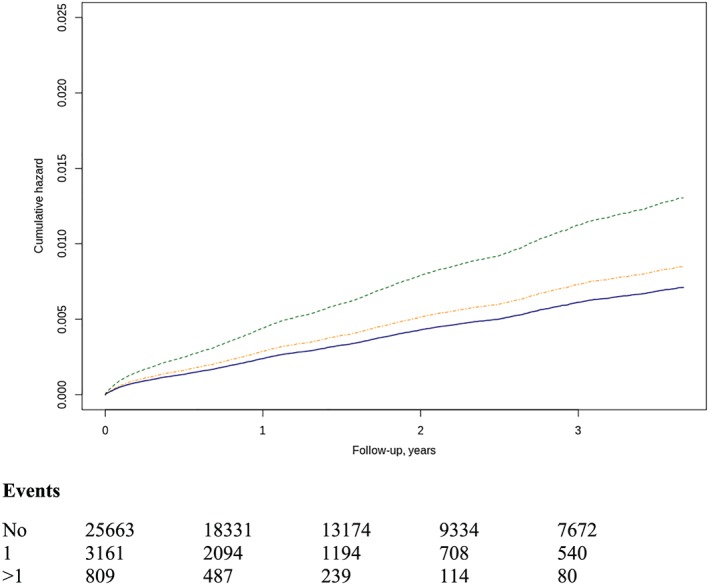
Risk of AHF hospital admissions by AKI number (A), one‐episode HR 1.19 (1.09–1.31), two or more episodes HR 1.84 (1.57–2.15). Line colours: blue (no AKI), orange (one AKI), and red (two or more AKI). Severity (B) of AKI episodes *risk* 1.17 (1.06–1.3), *injury* 1.80 (1.54–2.1), and *failure* 1.38 (1.09–1.75). Line colours as in *Figure* 1. AHF, acute heart failure; AKI, acute kidney injury; HR, hazard ratio.

A total of 3817 patients with AHF admission in the study period were recorded, with incidence of 38.4/100 HF patients/year. They were 3101 (81.2%) patients without AKI, 545 (14.3%) patients with one AKI episode, and 171 (4.5%) patients with two or more episodes. According to the AKI severity, AKI risk was present in 472 (12.4%), *injury* in 171 (4.5%), and *failure* in 73 (1.9%). The number of AKI episodes [one HR 1.05 (0.98–1.13); two or more HR 2.01 (1.79–2.25)] and severity [*risk* HR 1.05 (0.97–1.04); *injury* HR 1.41 (1.24–1.60); and *failure* HR 1.90 (1.64–2.20)] increases the risk of hospitalization, when adjusted by age, sex, CKD stage, hypertension, diabetes, ACEi/ARB, diuretics, number of visits to the specialist, and number of admissions (*Figure* *3*).

### 3.4 Acute kidney injury episodes and mortality

**Figure 4:**
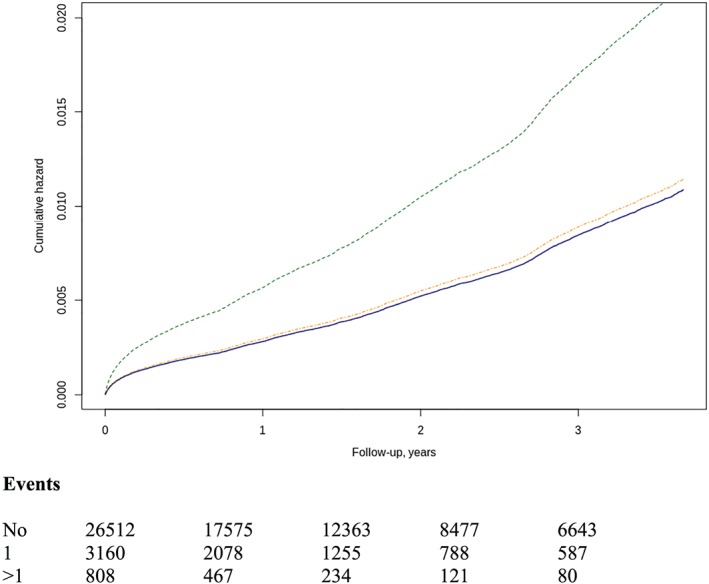
Risk of all‐cause mortality by AKI number (A) one‐episode HR 1.05 (0.98–1.13) and two or more episodes HR 2.01 (1.79–2.25). Line colours: blue (no AKI), orange (one AKI), and red (two or more AKI). Severity (B) of AKI episodes; HR for *risk* was 1.05 (95% CI 0.97–1.14), *injury* 1.41 (95% CI 1.24–1.60), and *failure* 1.90 (95% CI 1.64–2.20). Line colours as in *Figure* 1. AKI, acute kidney injury; HR, hazard ratio.

During the study period, a total of 10 560 deaths were recorded, with incidence of 9.3/100 HF patients/year, with 8951 (33.7%, incidence of 9/100 patients/year) of subjects without AKI episodes, 1180 (11.17%) of subjects with one episode, and 429 (4.06%) with two or more episodes. According to the severity of AKI, *risk* episodes were present in 992 (36.6%), *injury* in 367 (44.7%), and *failure* in 250 (57.2%). The number of episodes [one HR 1.05 (0.98–1.13); two or more HR 2.01 (1.79–2.25)] and severity [*risk* 1.05 (CI 0.97–1.14), *injury* 1.41 (CI 1.24–1.60), and *failure* 1.90 (CI 1.64–2.20)] increases mortality risk (*Figure* *4*). When the mortality risk was assessed in each of the eGFR groups according to the AKI severity, patients with eGFR of between 30 and 90 mL/min/1.73 m^2^ are those in whom the risk was graded by the severity of AKI episode. In patients with eGFR between 15 and 30 mL/min/1.73 m^2^, the risk of mortality is high in all subjects, with and without AKI episodes.

## 4 Discussion

In a large cohort of acute and chronic HF patients, AKI is a frequent condition in ~15% of patients with at least one episode, with an increasing incidence according to the eGFR group. AKI incidence and both number and severity of episodes increase the risk of hospital admission by AHF and all‐cause mortality.

The present study was conducted in a population from the Valencian Community territory, with an EHR associated with the public general‐practice setting that has a 92% coverage of the population living in the area. Every patient has a unique personal identification number, which guarantees the interoperability of the EHRs. Thus, administrative data, including all prescriptions and dispensation of subsidized treatments and hospitalization events, are linked to the database that integrates all the health care interventions and procedures that the patients received. Therefore, this study includes information on baseline risk factors and follow‐up for mortality and hospitalizations from adults with HF who had their serum creatinine repeatedly measured by the public health system during the study period.

Defining and classifying the sudden decrease in renal function are still a matter of debate with several criteria used, such as RIFLE,^15^ AKIN,^16^ KDIGO,^17^ and WRF.^18^ One study compared them in patients hospitalized due to acute heart decompensation to assess the benefits of using one or two of the AKI classification systems.^19^ The authors concluded that the potential advantages of the new classifications, AKIN and KDIGO, lie in the ability to identify those patients with more severe degrees of AKI who will go on to experience adverse events at 30 days and 1 year. However, the differences in terms of predictive abilities were only marginal.^19^ Diagnosis and classification of AKI were performed based on the RIFLE scale on the changes in serum creatinine, without the criteria that include urine output, owing to the characteristics of the database that do not record specific data from the hospitalization period or the ambulant control.

In patients with HF, AKI is a frequent event in which the haemodynamic status, low cardiac output or congestive status, and the impact of drugs, mainly diuretics and renin–angiotensin system blockade, are relevant factors. As it is expected, the incidence increases in patients with eGFR < 60 mL/min/1.73 m^2^ owing to the lower renal functional reserve produced by the HF itself or owing to age and co‐morbidities that increase the risk of renal functional deterioration. The more reduced eGFR increases not only the incidence but also the severity and reduces the lag period of time free of AKI. These data are in agreement with those of previous studies performed in AHF subjects, but no information was available on the total HF population. Whether or not the incidence was higher in patients with HF with reduced ejection fraction than in those with HF with preserved ejection fraction, it is not possible to assess it in the present study because the left ventricular function was not properly recorded in a great proportion of patients.

The importance of co‐morbidities in the evolution and prognosis of HF has been emphasized,^20^ and several studies have tried to identify factors related with hospitalization and prognosis.^21^, ^22^ Besides ischaemic heart disease, other factors such as non‐compliance to the prescribed treatments or diet, infections, arrhythmias, uncontrolled hypertension, anaemia, and renal dysfunction are among the most common reasons for AHF, progression, and mortality. The relevance and impact of AKI in terms of prognostic value for hospital admission by AHF or mortality have been investigated in the present study. Hospital admissions were considered only for those due to AHF avoiding total hospitalizations because patients with HF could have admission due to decompensation or other deadly co‐morbidities. AKI increases risk of hospitalization and mortality, indicating the more fragile status of the patient.

The limitations and strengths of the present study should be considered. First is with regards the definition of AKI. While we recognized the utility of AKI definition in epidemiology and clinical research arenas, it is not sufficiently validated for use in the diagnosis and clinical management of patients. The study does not identify the cause of mortality, whether it is cardiovascular or not, although the AKI represents a status of fragility that can alert about the very high risk of all‐cause mortality. Moreover, the lack of assessment of natriuretic peptides and left ventricular ejection fraction precluded to better dissect the effect of AKI across the different phenotypes of HF. The large sample size and the average follow‐up are useful to properly assess the significance of changes in renal function in terms of prognosis.

In conclusion, the study described the prognostic value of sudden renal function decline beyond the episode of AKI and pointed to the patients with more future risk who require review of treatment and more careful follow‐up. As the problem of hospitalization for AHF is expected to increase, we have to design strategies of management in the future. Further research is needed to find which is the best plan or site of management for these patients, taking into account that this can vary according to the population or the organization of health systems.

## Conflicts of Interest

None declared.

## Funding

This work was supported by the BigData@Heart (IMI2‐FPP116074‐2), BigMedilytics (ICT‐15‐780495), and CIBERObn Carlos III Health Institute (PI16/01402).
